# Prdx5 in the Regulation of Tuberous Sclerosis Complex Mutation-Induced Signaling Mechanisms

**DOI:** 10.3390/cells12131713

**Published:** 2023-06-24

**Authors:** Judit Bovari-Biri, ElHusseiny Mohamed Mahmoud Abdelwahab, Kitti Garai, Judit E. Pongracz

**Affiliations:** Department of Pharmaceutical Biotechnology, Faculty of Pharmacy, University of Pecs, 2. Rokus Str, H-7624 Pecs, Hungary

**Keywords:** TSC mutation, TrxR, Prdx5, mitochondria, rapamycin, auranofin, ER stress

## Abstract

(1) Background: Tuberous sclerosis complex (TSC) mutations directly affect mTORC activity and, as a result, protein synthesis. In several cancer types, TSC mutation is part of the driver mutation panel. TSC mutations have been associated with mitochondrial dysfunction, tolerance to reactive oxygen species due to increased thioredoxin reductase (TrxR) enzyme activity, tolerance to endoplasmic reticulum (ER) stress, and apoptosis. The FDA-approved drug rapamycin is frequently used in clinical applications to inhibit protein synthesis in cancers. Recently, TrxR inhibitor auranofin has also been involved in clinical trials to investigate the anticancer efficacy of the combination treatment with rapamycin. We aimed to investigate the molecular background of the efficacy of such drug combinations in treating neoplasia modulated by TSC mutations. (2) Methods: TSC2 mutant and TSC2 wild-type (WT) cell lines were exposed to rapamycin and auranofin in either mono- or combination treatment. Mitochondrial membrane potential, TrxR enzyme activity, stress protein array, mRNA and protein levels were investigated via cell proliferation assay, electron microscopy, etc. (3) Results: Auranofin and rapamycin normalized mitochondrial membrane potential and reduced proliferation capacity of TSC2 mutant cells. Database analysis identified peroxiredoxin 5 (Prdx5) as the joint target of auranofin and rapamycin. The auranofin and the combination of the two drugs reduced Prdx5 levels. The combination treatment increased the expression of heat shock protein 70, a cellular ER stress marker. (4) Conclusions: After extensive analyses, Prdx5 was identified as a shared target of the two drugs. The decreased Prdx5 protein level and the inhibition of both TrxR and mTOR by rapamycin and auranofin in the combination treatment made ER stress-induced cell death possible in TSC2 mutant cells.

## 1. Introduction

**Figure 1:**
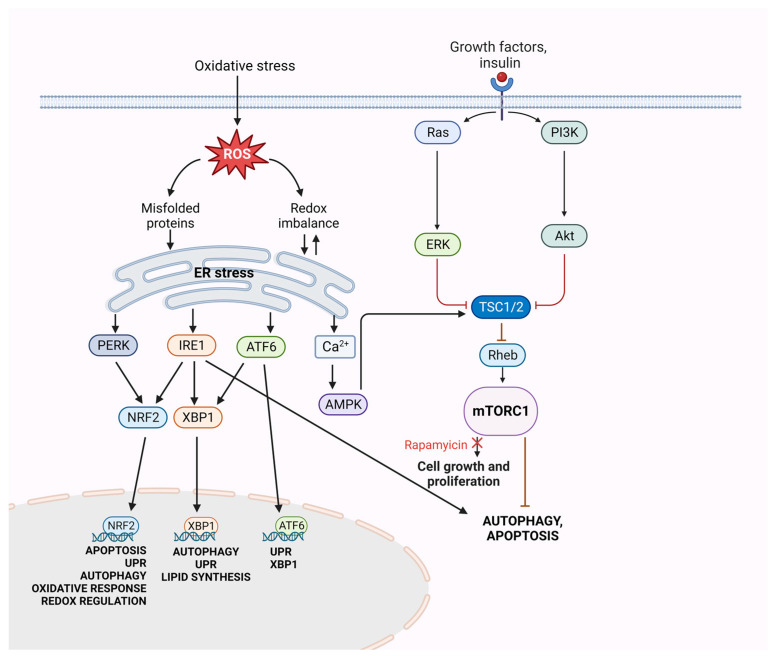
Signaling pathways associated with mTOR activity.

While oncogenic driver mutations serve as diagnostic markers as well as therapeutic targets, metabolic changes are frequently investigated and incorporated into disease management. One of the characteristic mutations that is found in many cancer types is associated with the mammalian target of rapamycin, i.e., mTOR. mTOR plays a key role in cell growth and proliferation via the catalytic subunit of two protein kinase complexes: mTOR complexes 1 and 2 (mTORC1/2). mTORC1 signaling is activated by several oncogenic signaling pathways and becomes hyperactive in most cancers, including giant cell astrocytoma [1], renal cell carcinoma [2,3], endometrial endometrioid adenocarcinoma [4], high-grade ovarian serous adenocarcinoma [4], rectal cancer [5], breast cancer [6], and in various subtypes of non-small-cell lung cancer [7,8] including lung adeno- [4] and squamous cell carcinomas [9,10]. Physiologically, the inhibitors of mTORCs are Tuberous Sclerosis Complex 1 (TSC1) and TSC2 proteins, which are important tumor suppressors that inhibit cell growth (Figure 1).

Genetic mutations or alterations in TSC1/2 have an important role not only in various cancers but also in some slow-growing neoplasms like lymphangioleiomyomatosis (LAM) [11] or angiomyolipoma (AML) [12]. Therefore, the inhibition of mTOR is an important drug target in cancer chemotherapy, and if possible, rapamycin is incorporated into the treatment regimen [13,14,15]. 

It is well known from previous studies that the continuous growth of cancer cells enabled by constant mTORC activity leads to alterations in their cellular metabolism. The high proliferation rates of cancer cells require increased protein production and, as a result, higher activity of the endoplasmic reticulum (ER). Increased demand on protein folding, assembly, and transport can induce physiological ER stress [16]. ER stress can be caused by both a lack and excess of nutrition, leading to reactive oxygen species (ROS) production [17]. Three ER stress signaling pathways have been identified: the inositol-requiring enzyme 1α (IRE1α), activating transcription factor 6 (ATF6), and pancreatic ER kinase-like ER kinase (PERK). IRE1α and its downstream signaling target X-box binding protein (XBP1), as well as PERK/eukaryotic initiation factor 2α (eIF2α)/ATF4, contribute to cancer progression [18] (Figure 1). 

Previous studies have associated TSC mutation-induced mTORC activation with mitochondrial malfunction and reduced mitochondrial biogenesis [19]

Mitochondria and the ER are known to be interconnected via signal transduction, vesicle transport, and membrane contact sites. Among other functions, these sites regulate mitochondrial quality control, lipid metabolism, calcium homeostasis, the unfolded protein response (UPR), and ER stress [20], indicating that an imbalance in mTOR signaling leads to complex malfunctions in ER stress and mitochondria-associated cell death and survival processes. 

In further support of such connections, the TSC mutation-induced deregulation of mTORC activation is linked to increased activity of the selenoprotein, i.e., thioredoxin reductase (TrxR) [21,22]. TrxR catalyzes thioredoxin reduction and consequently provides a defense mechanism against the oxidative damage caused by elevated ROS production [23]. The enzyme is upregulated in several types of cancers, where increased TrxR activity is associated with rapid tumor growth and a poor prognosis [24,25]. Similarly, increased TrxR activity was also detected in slow-growing LAM and AML, which are both driven by TSC mutation [19]. 

Not surprisingly, mTOR and TrxR activation, as well as ER stress, are targets for drug development to interfere with the adaptation of cancer cells to hypoxia and nutrient shortage and to develop drug resistance. Due to the molecular complexity of ER stress and autophagy, anticancer drugs did not have the desired effects due to an incomplete understanding of their role triggered in the ER [26]. However, patients whose treatment involved rapamycin for the inhibition of ribosomal protein S6 kinase beta-1 (S6K1), the downstream target of mTORC complex, experienced some beneficial effects, including slower tumor growth [27]. The orally administered gold-containing redox enzyme inhibitor auranofin [19], an inhibitor of TrxR has increased cell death in some tumor cell cultures [27], leading to attempts to test its efficacy in clinical cases of fast-growing unresponsive tumors [28]. Unfortunately, individually targeting mTORC1 or TrxR with rapamycin or auranofin, respectively, did not dramatically increase progression-free survival. In a recent study, Xia et al. [29] used the rapamycin derivative everolimus and auranofin in combination to significantly suppress the tumor growth of HCT116 and SGC-7901 cancer cell lines and their xenografts in Balb/c mice. The mechanisms of cell death were also identified to be induced by oxidative stress, autophagy, and ER stress response [29]. The precise signaling mechanisms were difficult to decipher as the HCT116 cells were positive for KRAS and PIK3AC mutations [30] that affect the Akt/mTOR signaling pathway [31]. Clinical trials have also been attempted, wherein the combined use of the otherwise FDA-approved drugs did not always consider the molecular mechanism of action for the justification of the planned clinical trial. In the case of ovarian cancer [32], the clinical trial plan argued that auranofin and sirolimus (rapamycin) can be effective due to their immunosuppressive effects [28,33,34]. The initiators of the clinical trial argued that the combination of the two drugs would decrease the body’s immune response and may increase blood cell count, thus improving survival. The plan did not involve the specific inhibitory potential of the two drugs and consequent modulatory effects in cancer cell apoptosis. 

Based on the above information, we theorized that further investigation of the molecular mechanism of rapamycin and auranofin might facilitate more effective therapeutic application plans by selecting the appropriate cancers and patient populations. To exclude interference from the effects of other genetic drivers, the TSC2 mutant 621-102 and its TSC2 WT control 621-103 cell lines were used. 

## 2. Materials and Methods

### 2.1. Cell Cultures 

The 621-102 cell line carries a biallelic inactivation of TSC2 gene (TSC2 −/−), while its control—the 621-103 (TSC2 +/+) cell line—carries non-mutant TSC [35]. Both cell lines were a generous gift from Dr. Elisabeth Henske, Brigham and Women’s Hospital and Harvard Medical School, Boston, MA, USA. The cells were grown in Dulbecco’s modified Eagle’s medium supplemented with 10% fetal bovine serum and 100 U/mL of penicillin-streptomycin and were cultured at 37 °C in a humidified atmosphere with 5% CO_2_.

### 2.2. Treatments

The study was based on extensive preliminary research to identify the potentially suitable drug concentrations and incubation times. For the preliminary tests 621-103 (TSC2 +/+, WT) (S103) and 621-102 (TSC2 −/−, mutant) (S102) cell lines were used, and the results are summarized in Supplementary Figure S1.

Cells were cultured for 24 h before the designated drugs were added, then the cell cultures were incubated for a further 3 h or 48 h, respectively. Additionally, 10 nM rapamycin (InvivoGen, San Diego, CA, USA, Cat.no: tlrl-rap) and 0.75 µM auranofin (Sigma Aldrich, Cat.no: A6733, St. Louis, MO, USA), < 0.01% DMSO final concentration), in monotreatment or in combination, were added to the cells. Stocks were diluted in DMSO and stored at −20 °C in aliquots. After treatment cells were processed for various arrays.

### 2.3. Cell Proliferation Assay

Furthermore, 1 × 10^4^ cells/well 621-103 (TSC2 +/+, WT) and 2.5 × 10^4^ cells/well 621-102 (TSC2 −/−, mutant) cells were plated to an 8 well chamber slide and cultured for 24 h before adding rapamycin (10 nM) and/or auranofin (0.75 µM) for 48 h. To detect newly synthesized DNA, we used the Click-IT EdU Cell Proliferation Kit (Invitrogen, Waltham, MA, USA, Cat. no.: C10637) according to the manufacturer’s guidelines. Cells were incubated overnight in the presence of EdU solution. Images were captured by using a Zeiss LSM 710 confocal laser scanning microscope (Zeiss, Germany). The evaluation of digital images was performed by using ImageJ analysis software [36]. The proportion of EdU and DAPI positive cells was calculated on each image using equivalent magnification and equal area designation. Controls were TSC WT cells exposed only to DMSO (0.001%). 

### 2.4. Crystal Violet Assay

In total, 5000 cells per well of S103 (TSC WT) and S102 (TSC mutant) were seeded into 96 well plates 24 h prior to drug treatment. Cells were treated for 48 h with rapamycin (10 nM) and/or auranofin (0.25–1 µM). After 24 h or 48 h incubation, the cells were fixed by adding 25% glutaraldehyde (Electron Microscopy Sciences, Hatfield, PA, USA) for 10 min at room temperature and stained with 0.05% crystal violet (C3886, Sigma-Aldrich, St. Louis, MO, USA) for 15 min at room temperature on a vertical shaker. Cells were washed with water 3 times, and the plates were air-dried for 2 h. Methanol (200 µL) was added to each well and incubated for 10 min on a shaker at room temperature. Optical density was measured at 590 nm. Data were calculated in terms of % surviving attached cells (% crystal violet OD) compared to medium control-treated cells. Experiments were performed in quadruplicate 5 times [37].

### 2.5. Electron Microscopy

Cells were resuspended in 2.5% glutaraldehyde in 0.1 M sodium-cacodylate buffer (pH 7.4) for 24 h and rinsed in 0.1 M sodium-cacodylate buffer. The pellet was embedded in Spurr low-viscosity resin with ERL 4221 and cured at 70 °C for 16 h. To perform transmission electron microscopy (TEM), 90 nm thick sections were stained with Reynolds lead-citrate and alcoholic uranyl acetate and examined using Jeol 1400 and Jeol 1200 transmission electron microscope (Jeol Ltd., Tokyo, Japan) at 80 kV. Images were captured using an integrated MegaView III digital camera (Olympus Soft Imaging Solutions GmbH; Munster, Germany) [19].

### 2.6. Staining of Functional Mitochondria

The control and rapamycin- (10 nM) and/or auranofin- (0.75 µM) treated cells were incubated at 37 °C in a CO_2_ incubator (5% CO_2_) for 1 h in the presence of 100 nmol/L MitoBright Green mitochondrial staining (Dojindo Molecular Technologies, Inc., Kumamoto, Japan, Cat.no: MT-06). MitoBright Green fluorescent dye accumulated and was retained in healthy mitochondria based on membrane potential dependency and covalent bonding to proteins. Stained cells were observed, and images were captured using Zeiss LSM 710 confocal laser scanning microscope (Zeiss, Germany). The evaluation of digital images was performed using ImageJ analysis software [38].

### 2.7. Thioredoxin Reductase (TrxR) Assay

Cells were seeded (3 × 10^4^ cells/well) in a 24-well plate and cultured for 24 h before adding drug treatments. After 48 h incubation with rapamycin (10 nM) and/or auranofin (0.75 µM), TrxR enzyme activity was measured in both cell lines using a colorimetric thioredoxin reductase assay kit (Abcam, London, UK, Cat.no: ab 83463) according to the manufacturer’s instructions. The colorimetric signal was detected using a Perkin Elmer Enspire Multiplate Reader (Perkin Elmer, Waltham, MA, USA) [39]. 

### 2.8. Ingenuity Pathway Analysis

The Ingenuity Pathway Analysis (IPA) (http://analysis.ingenuity.com, accessed on 5 April 2023, Ingenuity Systems; Qiagen N.V., Venlo, The Netherlands) was used for molecular pathway analysis. The interaction network analysis was performed to show the potential target molecules of auranofin and rapamycin in the dataset. IPA uses a network generation algorithm to segment the network map between molecules into multiple networks. In the next step, Molecule Activity Predictor (MAP) analysis filter was applied to predict the role of drugs in molecular activation or inhibition [40].

### 2.9. Taqman Assay

Following the collection of the treated cells in RA1 lysis buffer, total RNA isolation protocol was performed according to the protocol of Nucleospin RNA Isolation Kit (Macherey Nagel, Germany, Cat. no: 740955.250). Applied Biosystems High-Capacity cDNA Reverse Transcription Kit was used in cDNA writing process, according to the manufacturer’s protocol. In order to run Taqman (Thermo Fisher Scientific, Waltham, MA, USA) assays, Quantstudio 12K Flex Real-Time PCR System (Thermo Fisher Scientific) was used. Gene expression was normalized to GAPDH housekeeping gene (see Table 1 for a detailed assay list). The measurements were performed in triplicate. Data evaluation was carried out using the comparative ddCt method.

### 2.10. Cell Collection for ELISA and Cell Stress Array 

Six well plates were seeded with 2 × 10^5^ cells/well. The cells were cultured for 24 h before treatment. After 3 h incubation with rapamycin (10 nM) and/or auranofin (0.75 µM), the cells were collected and lysed in ice-cold RIPA-buffer supplemented with a protease inhibitor cocktail (Merck, Germany, Cat. no.: P8340) for 30 min at 4 °C and centrifuged for 20 min at 16,000× *g*, at 4 °C [41].

### 2.11. Prdx5 Protein Quantification-ELISA

Human Peroxiredoxin 5 ELISA Kit (Abcam, Cambridge, UK, Cat. no: ab283988) was used to detect protein concentration of Prdx5. The measurement protocol was created according to the manufacturer’s guidelines. The absorbance was measured using Perkin Elmer Enspire Multiplate Reader (Perkin Elmer, Waltham, MA, USA) [39,42].

### 2.12. Determination of Cell Stress-Related Proteins

Equal amounts of protein (15 μg) were applied for incubation with the array membranes according to the manufacturer’s protocol. The relative levels of cell stress-related proteins were detected using the Proteome Profiler Array, Human Cell Stress Array Kit (RnDSystem, USA, MN, Cat. No: ARY018) [43]. The luminescent signal was detected using an ImageQuant LAS-4000 imager (GE Healthcare Life Sciences, Chicago, IL, USA). Densitometric analyses of the arrays were performed using ImageJ software (ImageJ, NIH, Bethesda, MD, USA) and its Protein Array Analyzer plugin. The signal intensities of the proteins were normalized to the average of the three reference controls. 

### 2.13. Statistical Analysis

Graphs were generated, and statistical analysis was performed using the GraphPad Prism8 software. Data are presented as mean ± standard error of the mean of three replicates. Statistical analysis was performed using two-way ANOVA and paired *T*-test. *p* < 0.0332 was considered significant and marked by an asterisk (*p* < 0.0021 (**), *p* < 0.0002 (***) and *p* < 0.0001(****)).

## 3. Results

### 3.1. Main Manifestations of TSC Mutation in the 621-S102 Cell Line

**Figure 2:**
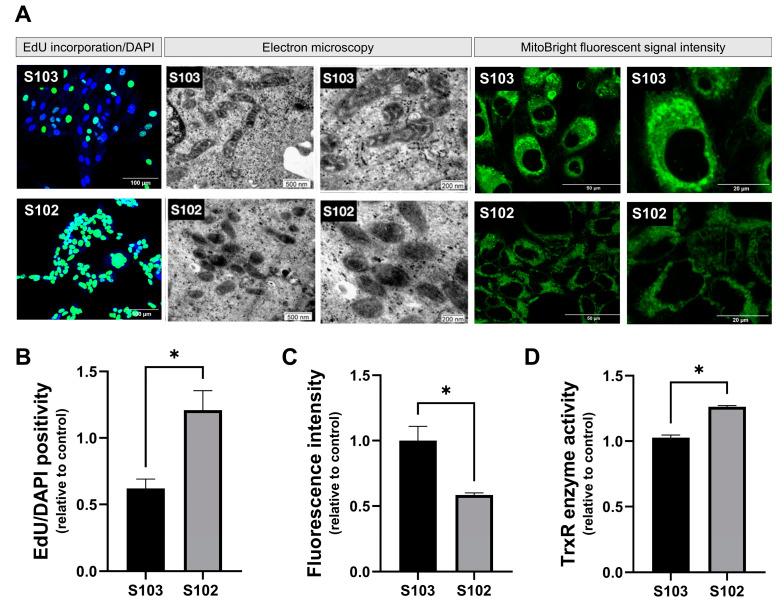
(**A**) Images show the differences between TSC WT (S103) and TSC mutant (S102) cells. In the first two images, EdU/DAPI staining represents the increased proliferative capacity of the mutant cells compared to the normal, control cell line. Incorporated EdU was visualized by Alexa 488, and the nucleus staining was performed by Hoechst 33342. Size bar: 100 µm. The second four images display the electron microscopic images of mitochondria in two different magnifications (Size bars: 500 nm and 200 nm). The last four images indicate cells after the accumulation of a membrane-potential-dependent dye (MitoBright Green) in functional mitochondria. Size bar: 200 µm. (**B**) Quantification of EdU incorporation-based cell proliferation of TSC mutant S102 cells was compared to the control TSC WT S103 cell line (n = 3). Statistically significant values (±SEM) are marked with an asterisk (*) (*p* < 0.0332), and statistical significance was calculated with paired *T*-test. (**C**) Quantified values of detected MitoBrigh Green fluorescence intensity in the two different cell lines (n = 3). Statistically significant values (±SEM) are marked with an asterisk (*) (*p* < 0.0332), and significance was calculated with paired *T*-test. (**D**) TrXR activity of TSC WT S103 and TSC mutant S102 cells (one representative graph of n = 3). Statistically significant values (±SEM) are marked with asterisk (*) (*p* < 0.0332).

To confirm that mitochondrial morphology, mTOR, and TrxR activity [19] are characteristic to TSC mutant cells, the proliferation capacity was determined in TSC WT S103 cells and in TSC deficient S102 (Figure 2). The 5-Ethynyl-2′-deoxyuridine (EdU) is a thymidine analog, which incorporates into the DNA of dividing cells. EdU incorporation into the TSC deficient S102 cell line confirmed active DNA synthesis and therefore increased proliferative capacity compared to the TSC WT S103 cell line (Figure 2A,B). The higher proliferative capacity of the TSC mutant S102 cell line correlated with altered mitochondrial morphology, which was detected via electron microscopy (Figure 2A), and reduced mitochondrial function by MitoBright Green (Figure 2A,C). As MitoBright Green accumulates in the functional mitochondria, the intensity of the fluorescence signal depends strongly on membrane potential. This is how the presence of dysfunctional mitochondria in TSC mutant S102 cells was confirmed. In parallel, TrxR’s regulatory activity in terms of ROS production was also increased in the TSC mutant S102 cells (Figure 2D). 

**Figure 3:**
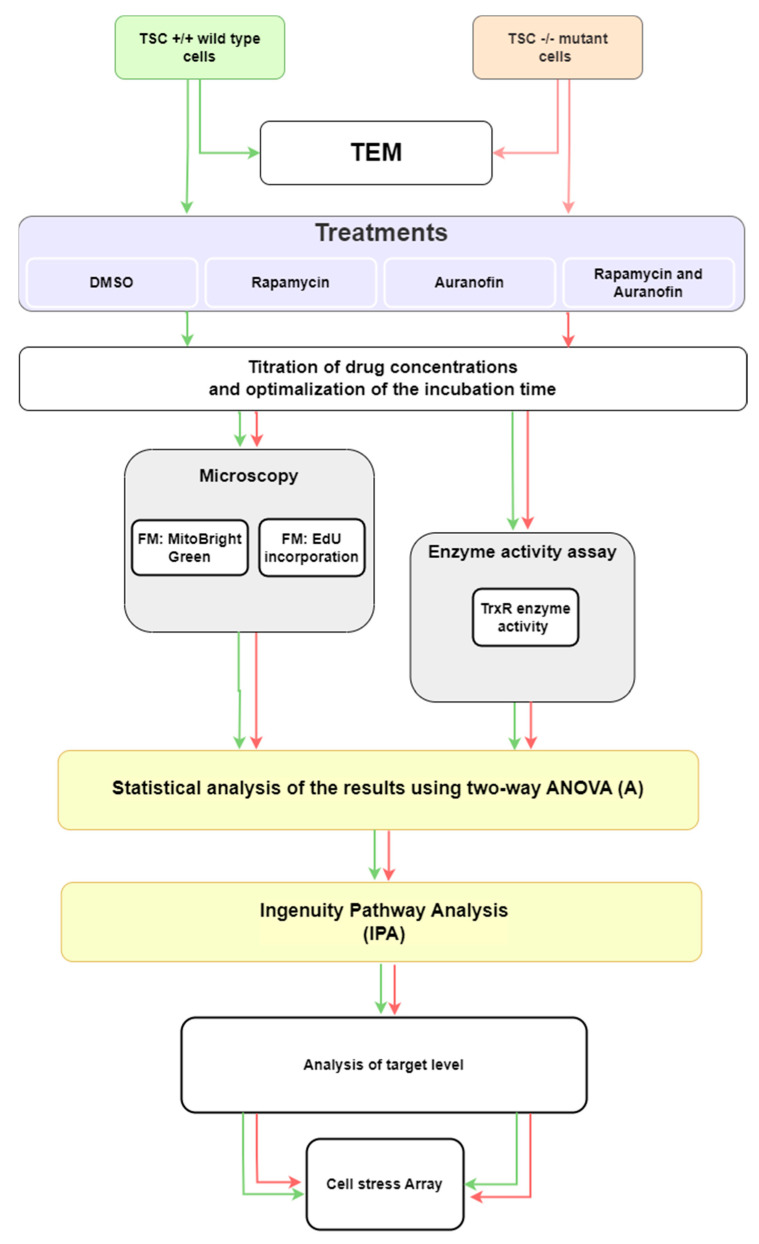
Experimental design.

Based on the natural characteristics of TSC2 mutation-induced physiological changes in mitochondrial morphology, membrane potential, and TrxR activity, the experimental design created to test the effects of rapamycin and auranofin was devised, and it is summarized in Figure 3.

### 3.2. Effects of mTORC and TrxR Inhibition on Cellular Proliferation and Mitochondria

For clinical intervention with TSC mutation-induced mTORC and TrxR activity, two FDA-approved drugs were used: the mTOR pathway inhibitor rapamycin [44] and the TrxR inhibitor auranofin [45]. To investigate how proliferation is affected by the cytostatic rapamycin and the redox enzyme inhibitor auranofin, a cellular proliferation assay was performed in the presence of the two drugs in mono- and in combination treatment. The concentrations used during the studies were selected following initial drug titration experiments (Supplementary Figure S1). Based on the titration results, 10 nM rapamycin and 0.75 µM auranofin were selected for further study. 

**Figure 4:**
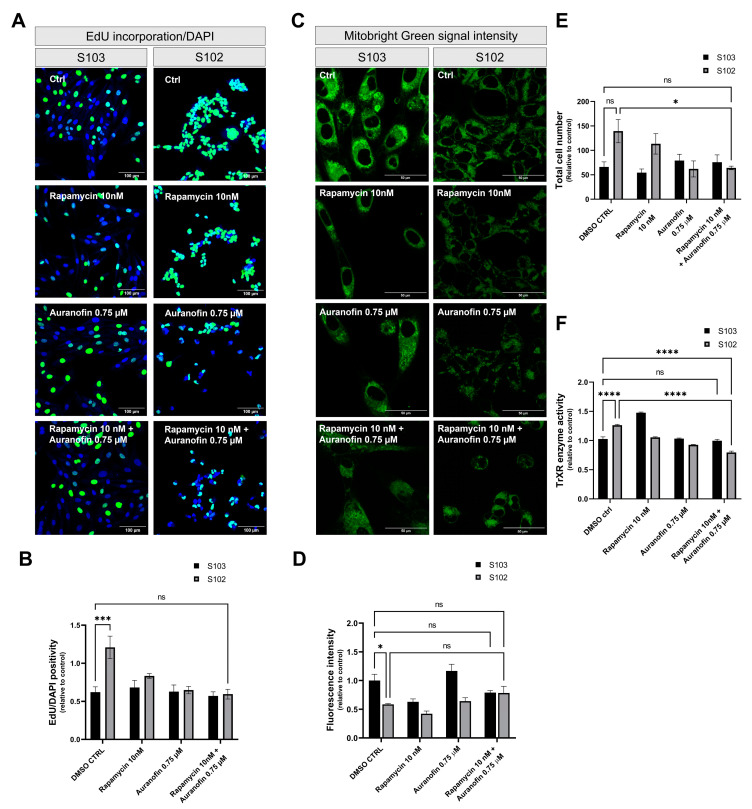
The effects of rapamycin (10 nM) and/or auranofin (0.75 µM) in TSC mutant (S102) and TSC WT (S103) cell lines. (**A**) Fluorescent microscopic analysis of cell proliferation in rapamycin (10 nM) and/or auranofin (0.75 µM) treated cells compared to DMSO (0.001%) control cells. Incorporated EdU was visualized by Alexa 488; nucleus staining was performed by Hoechst 33342. Size bar: 100 µm. (**B**) Quantification of EdU incorporation-based cell proliferation of TSC mutant (S102) cells was compared to the control TSC WT (S103) cell line (n = 4). The numeric values of EdU were compared to the DMSO (0.001%) control cell line (S103). Statistically significant values (±SEM) are marked by an asterisk (***) (*p* < 0.0002). (**C**) Staining of functional mitochondria based on membrane potential-dependent dye (MitoBright Green) accumulation. Size bar: 200 µm; (**D**) Quantified values of detected fluorescence intensity in case of various treatments in the two different cell lines. (n = 3). The MitoBright Green positivity values were compared to the DMSO- (0.001%) treated control cell line TSC WT (S103). Statistically significant values (±SEM) are marked with an asterisk (*) (*p* < 0.0332). (**E**) Quantified values of the total cell numbers after various treatments in the two different cell lines (n = 3). The cell number values were compared to the DMSO- (0.001%) treated control cell line TSC WT S103. Statistically significant values (±SEM) are marked with an asterisk (*) (*p* < 0.0332). (**F**) TrXR activity of TSC WT S103 and TSC mutant S102 cells after rapamycin (10 nM) and/or auranofin (0.75 µM) treatment (n = 3). Data were calculated and compared to the untreated TSC WT S103 control cell line. Statistically significant values (±SEM) are marked with asterisk (****) (*p* < 0.0001). Statistically non-significant values are labeled as ns.

Both drugs reduced cellular proliferation in mono treatment (Figure 4A,B) and in combination treatment, although the effect of rapamycin and auranofin was not additive (Figure 4A,B), indicating action via separate pathways. EdU incorporation was significantly reduced in rapamycin- and auranofin-treated TSC mutant S102 cells compared to the DMSO-treated TSC mutant S102 controls. Combined rapamycin and auranofin treatment of TSC WT S103 cells did not differ significantly from the DMSO-treated TSC WT S103 controls (Figure 4B), indicating that cells with normal TSC genes were less sensitive to the two drugs.

To investigate the effects of rapamycin (10 nM) and/or auranofin (0.75 µM) on mitochondrial membrane potential, MitoBright Green staining was performed. In the untreated TSC mutant S102 cell line, the fluorescence signal was significantly lower than in the TSC WT S103 control cells, indicating reduced mitochondrial function (Figure 4C). Rapamycin (10 nM) monotreatment induced reduction (Figure 4C,D), while auranofin (0.75 µM) slightly increased mitochondrial membrane potential (Figure 4C,D) compared to rapamycin treatment. Rapamycin (10 nM) and auranofin (0.75 µM) combination treatment almost normalized mitochondrial membrane potential, facilitating the survival of certain cells (Figure 4C,D) while triggering apoptosis in the majority of TSC mutant cells. 

The analysis of the total cell number following rapamycin (10 nM) and/or auranofin (0.75 µM) treatment supports the above results as the cell number did not change dramatically after rapamycin monotreatment in contrast to auranofin monotreatment, where significant reduction was detected in cell numbers (Figure 4E). 

To investigate TrxR activity, the cell lines were treated with auranofin (0.75 µM). Treatment involving auranofin (0.75 µM) and mono combination treatments involving auranofin (0.75 µM) and rapamycin (10 nM) (Figure 4F) induced decreased TrxR activity compared to untreated TSC mutant S102, and this decrease was observed to be even lower when compared to TSC WT S103 controls. This observation is important, as it supports the notion that TSC WT S103 cells sustain less damage due to increased ROS levels than TSC mutant S102 cells. This increase in ROS levels is caused by reduced TrxR activity. 

Based on the above results, as well as the existing literature [46], a connection to an ER-dependent signaling mechanism was predicted. 

### 3.3. The Shared Target of Auranofin and Rapamycin Signaling–Prdx5 

**Figure 5:**
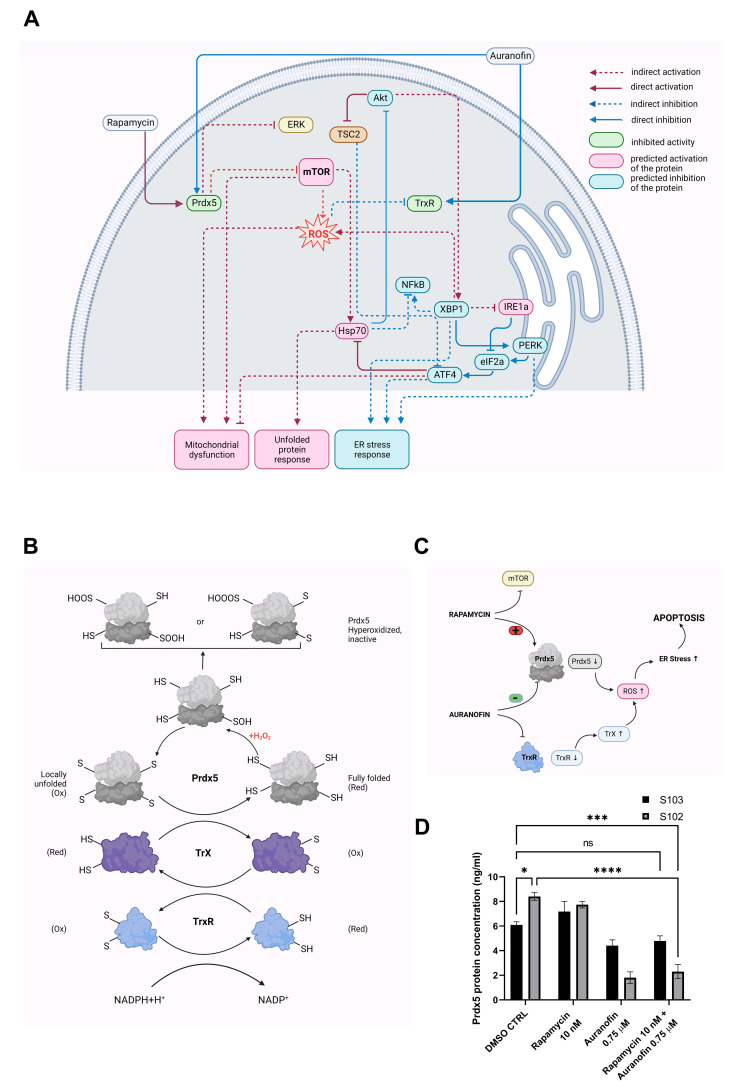
Prdx5 enzyme is a shared target molecule of rapamycin and auranofin. (**A**) Summary of IPA, Molecular Activity Prediction analysis. (**B**) Schematic figure depicts the role of Prdx5 and TrxR redox enzyme in oxidative processes. (**C**) The role of the common target molecule of rapamycin and auranofin treatments (identified by IPA) in ROS production and apoptosis induction. (**D**) Prdx5 protein expression level of TSC WT (S103) and TSC mutant (S102) cells after rapamycin (10 nM) and/or auranofin (0.75 µM) treatment (n = 3). Data were calculated and compared to the untreated TSC WT control cell line (S103). Statistically significant values (±SEM) are marked with asterisks: (*) *p* < 0.0332; (*p* < 0.0002 (***), and *p* < 0.0001 (****). Statistically non-significant values are labeled as ns.

Apart from their direct targets TrxR for auranofin and mTOR for rapamycin, a shared target or target mechanism was suspected due to the modified drug effect in the combination treatment for the TSC mutant cell lines. The target search was performed by the Ingenuity Pathway Analysis (IPA) database using the Drug target and the Molecular Activity Predictor analysis filters. The analysis identified Prdx5 as a joint target of the two drugs (Figure 5A). Prdx5 is a thioredoxin peroxidase enzyme expressed in various tissues at different levels [47]. It is distributed at a subcellular level in mitochondria, peroxisomes, cytosol, and the nucleus [48]. Prdx5 acts via cytosolic or mitochondrial thioredoxins and has a central role in reducing alkyl hydroperoxides and peroxynitrite. The IPA database highlighted the possibility that Prdx5 is activated by rapamycin and inhibited by auranofin (Figure 5B). Additionally, a previous study demonstrated that Prdx5 has an important role in the crosstalk between mitochondria and the ER [31] (Figure 5C).

To test how auranofin and rapamycin affect Prdx5, both a Taqman assay (Supplementary Figure S2) and a sandwich ELISA was performed (Figure 5D). 

While Prdx5 mRNA changes were not significant, Prdx5 protein levels changed significantly after treatment. In terms of the untreated cell lines, Prdx5 protein expression was significantly higher in the TSC mutant S102 cell line than in the TSC WT S103 control cells, indicating the existence of an additional protective system against elevated ROS production in TSC mutant cells. Rapamycin (10 nM) monotreatment increased Prdx5 levels in the TSC WT S103 cells but had no effect in the TSC mutant S102 cells (Figure 5B). In contrast, auranofin (0.75 µM) monotreatment reduced the Prdx5 protein levels in both the TSC mutant S102 and TSC WT S103 cells. The same effect was detected after rapamycin and auranofin combination treatment, but in the TSC mutant S102 cells, Prdx5 protein levels were reduced more drastically than in the TSC WT S103 controls (Figure 5D). Based on the literature [46] as well as our results, we can confidently state that increased ROS production is linked to ER stress. 

### 3.4. Prdx5 and ER Stress

**Figure 6:**
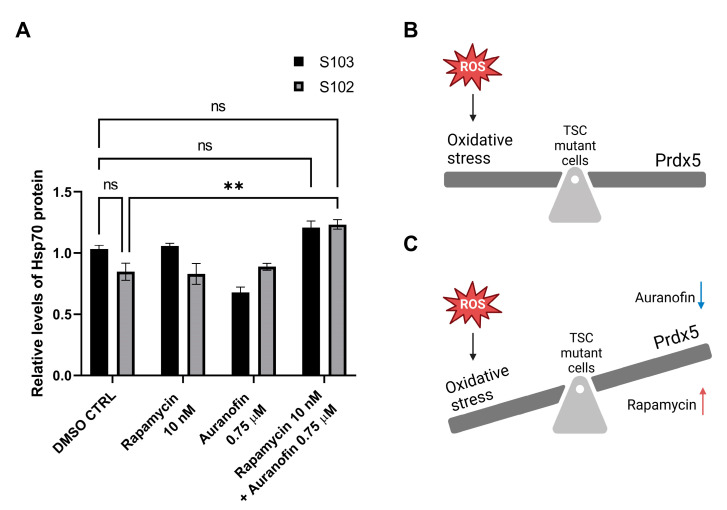
(**A**) Hsp70 protein levels detected using a Human Proteome Profiler Cell Stress Array Kit following rapamycin- and/or auranofin-based treatment of TSC WT (S103) and TSC mutant (S102) cells. Statistically significant values (±SEM) are marked with (*p* < 0.0021 (**). Statistically non-significant values are labeled as ns. (**B**) The schematic figure shows increased ROS production as a result of mTOR hyperactivation induced by TSC2 mutation. The cellular metabolism counteracts elevated ROS production by increasing the level of various antioxidant enzymes. (**C**) The schematic figure depicts how the imbalance between increased ROS and deregulated antioxidant enzyme concentration affects TSC mutant cells (due to rapamycin and auranofin treatment). The applied drug combination results in the inhibition of TrxR activity and decreased Prdx5 protein levels, which can induce extreme ROS spill-over and increased cell death among TSC mutant cells.

To find the link between Prdx5 and ER stress signaling, 25 cellular stress-related proteins were screened using a Cell Stress Array, Human Proteome Profiler Kit (Figure 6A) (Supplementary Figure S4). The control and rapamycin- and auranofin-treated cell lines were screened 3 h after drug treatments. 

We detected the upregulation of the Heat Shock Protein (HSP) 70 protein after combination treatment with rapamycin (10 nM) and auranofin (0.75 µM). The level of HSP 70 was increased both in TSC mutant S102 and in TSC WT S103 cell lines (Figure 6, Supplementary Figure S3). Hsp 70 is an ER stress marker that plays a central role in the unfolded protein response (UPR), a well-known cellular stress response mechanism. 

## 4. Discussion

TSC mutant cells demonstrate increased proliferation ability, reduced mitochondrial membrane potential, increased TrxR activity [21,49], and aberrant mitochondrial biogenesis [50]. mTOR regulates mitochondrial activity [51] and plays a central role in the relative balance of ATP generation between mitochondrial and non-mitochondrial sources. mTORC1 can induce changes in glucose metabolism and generates a shift from oxidative phosphorylation (OxPhos) to glycolysis [52]. In all cellular events, such as cell division, migration, proliferation, regulation of cell size, and autophagy, there is a great need for an adequate amount of energy. The regulation of cellular events and metabolism, including respiration and energy production, is strict. In cancer cells, the cellular events have an increased need for ATP to cover the elevated proliferation rate and migration capacity. The larger part of ATP in cancer cells is produced via the OxPhos mechanism [53]. The metabolic pathway OxPhos is an in which an enzymatic process occurs to oxidize nutrients for the purposes of yielding high levels of ATP. However, in numerous cases, cancer cells produce ATP via anaerobic fermentation, with higher glycolysis levels even in the presence of oxygen (Warburg effect) [54,55]. The Warburg effect has also been detected in TSC-deficient cell lines and TSC mutation-driven neoplasms like LAM and AML [51]. The “Warburg effect” during respiration leads to a higher amount of ROS and a decrease in antioxidative capacity in the affected cells, which results in mitochondrial damage [55]. The cell in the state of elevated ROS production increases its ROS scavenging system via NADPH oxidation and the activation of TrxR. Ordinarily, the antioxidant matrix NADPH reductases (glutathione reductases and TrxRs [56]) can all generate H_2_O_2_ by leaking electrons from their reduced flavoprotein to O_2_. The generation of this mitochondrial ROS spill-over can induce oxidative injury and cause extreme mitochondrial damage. Redox homeostasis, which controls ROS excess, is a critical element of cell survival. Numerous antioxidant molecules and cascades are involved in the process, including the PrdX proteins. The Prdx family is composed of thiol-dependent peroxidase enzymes that play a central role in the redox reactions of cells. Prdx5 is a thioredoxin peroxidase that does not require cofactors [57] and can be found in mitochondria, peroxisomes, cytosol, and the nucleus [48]. Its main function is to act as a cytoprotective antioxidant and reduce alkyl hydroperoxides and peroxynitrite via the oxidation of thioredoxins in the cytosol and in the mitochondria [58]. It is constitutively expressed in various mammalian cell lines and healthy tissues, although numerous transcription factors, such as nuclear factor-κβ (NF-κβ), the antioxidant response element (ARE), or the insulin response element (InRE) can induce changes in its expression. Increased levels of Prdx5 proteins were found in several aggressive cancer types, such as ovarian carcinoma [59], Hodgkin’s lymphoma [60], breast carcinoma [61], or thyroid cancer [62]. Reduced levels of Prdx5 enzymes have also been reported in adrenocortical carcinoma [62,63], indicating that several added factors and mechanisms regulate the outcome of cancer growth. As discussed above, in contrast to healthy cells, cancer cells are characterized by intensive ROS production due to their altered metabolism [64]. Hence, cancer cells protect themselves from the increased internal oxidative environment by increasing the levels of their antioxidant enzymes [64,65,66]. As ROS-induced cytotoxicity is the central element of chemo- and radiotherapies, the increased levels and activity of antioxidant enzymes—including Prdx5—play an important role in determining the chemo- and radio-resistance of cancer cells [67]. While the cell response depends on the antioxidant status of the cell, depletion of the antioxidant molecule Prdx5 can sensitize the cells to chemotherapy [57]. Studies show that cells with a silenced expression of Prdx5 are more vulnerable to oxidative damage and apoptosis [58,68]. According to previous studies, lower Prdx5 levels also correlate with slow tumor growth and reduced infiltration and metastasis capability [69]. In contrast to aggressive and fast-growing tumor types, such as lung adenocarcinoma, a previous study demonstrated variations in Prdx5 mRNA levels as prognostic factors in lung cancer, with low levels of Prdx5 correlating with poor overall survival [70]. The above study further supports that Prdx5 imbalance in connection with other cancer-specific molecular events, including various driver mutations and metabolic changes, determines its actual role in drug response and cancer cell survival (Figure 6). Additionally, the regulation of Prdx5 expression is complex, and apart from the already mentioned transcription factors (eg.AP-1, NF-kB, etc.), it also depends on ROS levels [71]. ROS-mediated hypomethylation of Prdx5 promoters leads to the activation of the nuclear factor erythroid 2-related factor 2 (NRF2), a transcription factor that regulates the cellular defense against toxic and oxidative insults through the expression of genes involved in oxidative stress response [72]. Results have revealed that approximately two thirds of NSCLC patients exhibit demethylation in the Prdx5 promoter region in a ROS-dependent manner, and this process is also related to tumor progression status (TNM stage). As not all lung cancer patients have shown such ROS-mediated hypomethylation, driver mutations also need to be considered in further studies to find additional regulatory mechanisms for Prdx5 expression. Further investigation is therefore needed to explain what metabolic malfunction or parallel driver mutation can lead to the overexpression, destabilization, or proteosome-mediated degradation of Prdx5.

Based on the above studies, it is hardly surprising that the race is ongoing to find a supplementary therapeutic intervention that not only targets specific mutations or immune regulation but also targets the metabolic pathways in cancer cells.

In TSC mutant S102 cells, the inhibition of TrxR and mTORC1 activity by auranofin and/or rapamycin, respectively, reduced proliferation capacity and increased mitochondrial membrane potential. In contrast, monotreatment with auranofin leads to massive cell death, indicating that the inhibited mechanisms that protect cancer cells from ROS overproduction are important elements of the neoplastic character. The regulation of Prdx5 is complex and is upregulated in TSC mutant S102 cells. The elevated Prdx5 levels can be decreased by rapamycin and auranofin combination treatment nearing its detection level in TSC WT cells. Prdx5 is a target of both rapamycin and auranofin. Rapamycin treatment alone induced no significant changes in Prdx5 levels in TSC mutant S102 cells, while in TSC WT S103 cells, Prdx5 expression levels slightly increased. In contrast, auranofin decreased Prdx5 expression in TSC mutant S102 cells, and using combined treatment with rapamycin led to a more moderate expression of the protein.

Based on previous studies and on our results, TSC mutation induces an imbalance in the antioxidant system of cancer cells, and the increased activity of TrxR and Prdx5 enzymes compensate for the elevated levels of ROS production and maintain an increased proliferative capacity, protecting the cells from ER stress-induced death. Auranofin can inhibit this preserved antioxidant status, leaving cancer cells unprotected against the elevated intracellular ROS levels and consequently against death (Figure 6). 

**Figure 7:**
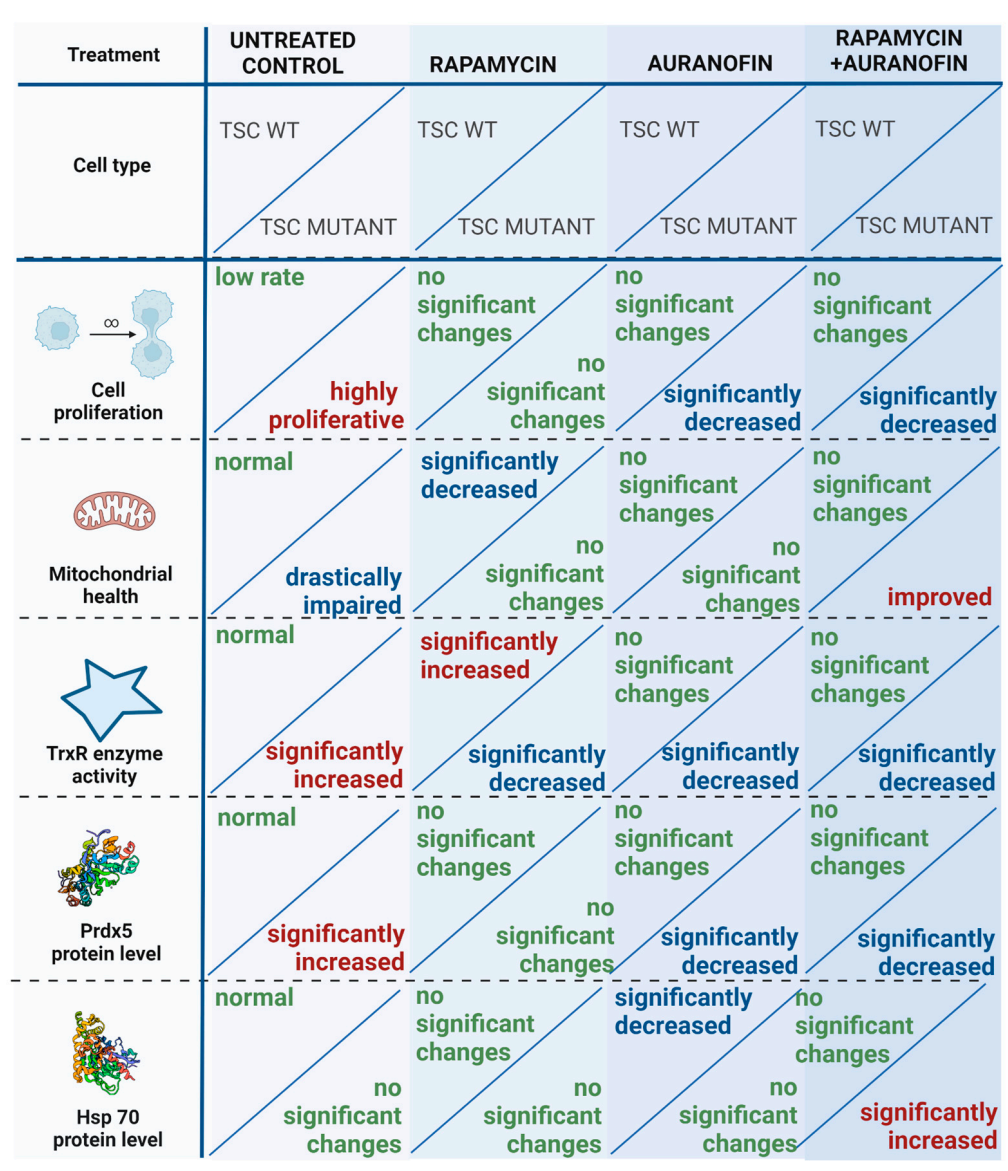
Summary figure of the effects of various treatments on TSC WT S103 and TSC mutant S102 cell lines. Changes in TSC WT S103 cells induced by various treatments were compared to the untreated control samples of TSC WT S103 cells, while in the case of TSC mutant S102 cells, the observed trends were compared to the results of untreated TSC mutant cells. The basic molecular levels and non-significant drug effects are noted in green, while increases and decreases are demarcated by being in red and blue, respectively.

As Prdx5 is a protein that provides crosstalk between the mitochondria and the ER [73], the significant upregulation of the ER stress protein Hsp 70 following auranofin and rapamycin combination treatment suggests that the ER and the UPR play an important role in ROS-induced cell death [74,75]. The well-known role of Hsp 70 is to prevent the formation of protein aggregates in times of elevated cellular stress. The accurately folded proteins enter into the Golgi complex, while misfolded proteins accumulate in the ER, tagged with Hsp 70 chaperon molecule. The complex of the unfolded or misfolded protein and the Hsp 70 molecule trigger the UPR-mediated cell death process [76,77]. Prxd5 can protect cells from such deaths, indicating a dual role of Prdx5 in these complex biochemical mechanisms. The direct effects of drugs on their physiological targets are summarized in Figure 7.

In summary, our experiments suggest that some patients affected by TSC mutation- regulated neoplasms might benefit from auranofin and rapamycin combination therapy, which could potentially prolong remission or slow disease progression via the normalization of Prdx5 levels and ER stress-dependent cellular death. The dosage of such a drug combination has to be carefully titrated for clinical application to various tumor types. Our data also indicate that, due to complexity of the mechanism, in vitro studies using three-dimensional primary cancer tissue organoids should precede any clinical trials.

## 5. Conclusions

In laboratory research, modulations in drug-induced mechanisms are easily studied, and discrepancies can be explained. While auranofin—similarly to rapamycin—monotreatment reduced the proliferation of TSC mutant S102 cells, it also affected the TSC WT S103 cells and the treatment was only tolerable for the control cell line when in combination with rapamycin. 

Although auranofin is an FDA-approved drug [78], it carries the risk of serious adverse reactions, including loose stool, skin irritation (20%), mouth ulcers (1–10%), proteinuria (5%), abdominal cramps, and even watery diarrhea [79,80]. Rapamycin (Sirolimus) is also an FDA-approved drug that is regularly used in cancer therapy [81]. Unfortunately, a significant number of patients develop severe side effects to rapamycin, which include edema, diarrhea, nephrotoxicity, dyspepsia, impaired wound healing, thrombocytopenia, stomatitis, or even hypercholesterolemia [11]. A cohort study showed that a lower serum level of rapamycin can be associated with fewer adverse events while mostly retaining drug efficacy [12,13]. Repurposing auranofin and rapamycin in combination therapy for the treatment of tumors with TSC mutations requires further investigation. Although both drugs are approved by the FDA, neither is approved for this particular combination; therefore, an investigation of their combined safety profile and biological effectiveness is required [82]. Due to the immunosuppressive effects of both drugs, such therapy for individual cancer patients should also be carefully timed to avoid interference with available immunotherapies.

## Figures and Tables

**Table 1:** Taqman assays.

Target Gene	Taqman Assay
GPDH	Assay ID:Hs02786624_g1
PRDX5	Assay ID: Hs01067165_g1

## Data Availability

Original data are available from the corresponding author upon request.

## Supplementary Materials

The following supporting information can be downloaded at: https://www.mdpi.com/article/10.3390/cells12131713/s1, Figure S1: Response of S102 and S103 cell lines to rapamycin and auranofin treatment. Figure S2: Prdx5 gene expression level of TSC WT (S103) and TSC mutant (S102) cells after rapamycin (10 nM) and/or auranofin (0.75 µM) treatment (n = 7). Figure S3: Protein expression levels of HSP70 protein of TSC WT (S103) and TSC mutant (S102) cells after rapamycin (10 nM) and/or auranofin (0.75 µM) treatment.

## References

[1] Ryall S., Tabori U., Hawkins C. (2020). Pediatric low-grade glioma in the era of molecular diagnostics. Acta Neuropathol. Commun..

[2] Miricescu D., Balan D.G., Tulin A., Stiru O., Vacaroiu I.A., Mihai D.A., Popa C.C., Papacocea R.I., Enyedi M., Sorin N.A. (2021). PI3K/AKT/mTOR signalling pathway involvement in renal cell carcinoma pathogenesis (Review). Exp. Ther. Med..

[3] Ghosh A.P., Sudarshan S. (2016). Genetics of renal cancer: Focus on MTOR. Aging.

[4] The AACR Project GENIE Consortium (2017). AACR Project GENIEpowering precision medicine through an international consortium. Cancer Discov..

[5] Slattery M.L., Herrick J.S., Lundgreen A., Fitzpatrick F.A., Curtin K., Wolff R.K. (2010). Genetic variation in a metabolic signaling pathway and colon and rectal cancer risk: mTOR, PTEN, STK11, RPKAA1, PRKAG2, TSC1, TSC2, PI3K and Akt1. Carcinogenesis.

[6] Jiang W.G., Sampson J., Martin T.A., Lee-Jones L., Watkins G., Douglas-Jones A., Mokbel K., Mansel R.E. (2005). Tuberin and hamartin are aberrantly expressed and linked to clinical outcome in human breast cancer: The role of promoter methylation of TSC genes. Eur. J. Cancer.

[7] Huang Q., Li F., Hu H., Fang Z., Gao Z., Xia G., Ng W.L., Khodadadi-Jamayran A., Chen T., Deng J. (2022). Loss of *TSC1/TSC2* sensitizes immune checkpoint blockade in non–small cell lung cancer. Sci. Adv..

[8] Lee Y.H., Do S.K., Lee S.Y., Kang H.G., Choi J.E., Hong M.J., Lee J.H., Lee E.B., Jeong J.Y., Shin K.M. (2019). TSC2 genetic variant and prognosis in non-small cell lung cancer after curative surgery. Thorac. Cancer.

[9] Mercurio L., Albanesi C., Madonna S. (2021). Recent Updates on the Involvement of PI3K/AKT/mTOR Molecular Cascade in the Pathogenesis of Hyperproliferative Skin Disorders. Front. Med..

[10] Tan F.H., Bai Y., Saintigny P., Darido C. (2019). mTOR Signalling in Head and Neck Cancer: Heads Up. Cells.

[11] Kristof A.S. (2010). mTOR signaling in lymphangioleiomyomatosis. Lymphat. Res. Biol..

[12] Kenerson H., Folpe A.L., Takayama T.K., Yeung R.S. (2007). Activation of the mTOR pathway in sporadic angiomyolipomas and other perivascular epithelioid cell neoplasms. Hum. Pathol..

[13] Voss M.H., Molina A.M., Motzer R.J. (2011). mTOR inhibitors in advanced renal cell carcinoma. Hematol. Oncol. Clin. N. Am..

[14] Darici S., Alkhaldi H., Horne G., Jørgensen H.G., Marmiroli S., Huang X. (2020). Targeting PI3K/Akt/mTOR in AML: Rationale and Clinical Evidence. J. Clin. Med..

[15] Tian T., Li X., Zhang J. (2019). mTOR Signaling in Cancer and mTOR Inhibitors in Solid Tumor Targeting Therapy. Int. J. Mol. Sci..

[16] Lee A.S. (2007). GRP78 induction in cancer: Therapeutic and prognostic implications. Cancer Res..

[17] Cao S.S., Kaufman R.J. (2014). Endoplasmic reticulum stress and oxidative stress in cell fate decision and human disease. Antioxid. Redox Signal..

[18] Xiong S., Chng W.J., Zhou J. (2021). Crosstalk between endoplasmic reticulum stress and oxidative stress: A dynamic duo in multiple myeloma. Cell. Mol. Life Sci..

[19] Abdelwahab E.M.M., Pal S., Kvell K., Sarosi V., Bai P., Rue R., Krymskaya V., McPhail D., Porter A., Pongracz J.E. (2019). Mitochondrial dysfunction is a key determinant of the rare disease lymphangioleiomyomatosis and provides a novel therapeutic target. Oncogene.

[20] Kato H., Nakajima S., Saito Y., Takahashi S., Katoh R., Kitamura M. (2012). mTORC1 serves ER stress-triggered apoptosis via selective activation of the IRE1-JNK pathway. Cell. Death Differ..

[21] Abdelwahab E.M.M., Bovari-Biri J., Smuk G., Fillinger J., McPhail D., Krymskaya V.P., Pongracz J.E. (2021). Activated p53 in the anti-apoptotic milieu of tuberous sclerosis gene mutation induced diseases leads to cell death if thioredoxin reductase is inhibited. Apoptosis.

[22] Bee J., Fuller S., Miller S., Johnson S.R. (2018). Lung function response and side effects to rapamycin for lymphangioleiomyomatosis: A prospective national cohort study. Thorax.

[23] Pannala V.R., Dash R.K. (2015). Mechanistic characterization of the thioredoxin system in the removal of hydrogen peroxide. Free. Radic. Biol. Med..

[24] Zhang W., Zheng X., Wang X. (2015). Oxidative stress measured by thioredoxin reductase level as potential biomarker for prostate cancer. Am. J. Cancer Res..

[25] Esen H., Erdi F., Kaya B., Feyzioglu B., Keskin F., Demir L.S. (2015). Tissue thioredoxin reductase-1 expression in astrocytomas of different grades. J. Neurooncol..

[26] Schleicher S.M., Moretti L., Varki V., Lu B. (2010). Progress in the unraveling of the endoplasmic reticulum stress/autophagy pathway and cancer: Implications for future therapeutic approaches. Drug. Resist. Updat..

[27] Wu X., Xie W., Wei W., Guo J. (2022). Beyond controlling cell size: Functional analyses of S6K in tumorigenesis. Cell. Death Dis..

[28] Gamberi T., Chiappetta G., Fiaschi T., Modesti A., Sorbi F., Magherini F. (2022). Upgrade of an old drug: Auranofin in innovative cancer therapies to overcome drug resistance and to increase drug effectiveness. Med. Res. Rev..

[29] Xia Y., Chen J., Yu Y., Wu F., Shen X., Qiu C., Zhang T., Hong L., Zheng P., Shao R. (2021). Compensatory combination of mTOR and TrxR inhibitors to cause oxidative stress and regression of tumors. Theranostics.

[30] Alves S., Castro L., Fernandes M.S., Francisco R., Castro P., Priault M., Chaves S.R., Moyer M.P., Oliveira C., Seruca R. (2015). Colorectal cancer-related mutant KRAS alleles function as positive regulators of autophagy. Oncotarget.

[31] Hao Y., Samuels Y., Li Q., Krokowski D., Guan B.J., Wang C., Jin Z., Dong B., Cao B., Feng X. (2016). Oncogenic PIK3CA mutations reprogram glutamine metabolism in colorectal cancer. Nat. Commun..

[32] Clinical Trial, I.N., Mayo Clinic (2023). Phase II Trial to Evaluate the Efficacy of Auranofin and Sirolimus in Serous Ovarian Cancer Patients with Recurrent Disease. NCT03456700.

[33] Rousselle B., Massot A., Privat M., Dondaine L., Trommenschlager A., Bouyer F., Bayardon J., Ghiringhelli F., Bettaieb A., Goze C. (2022). Conception and Evaluation of Fluorescent Phosphine-Gold Complexes: From Synthesis to in vivo Investigations. ChemMedChem.

[34] Dumont F.J., Su Q. (1996). Mechanism of action of the immunosuppressant rapamycin. Life Sci..

[35] Yue M., Pacheco G., Cheng T., Li J., Wang Y., Henske E.P., Schuger L. (2016). Evidence Supporting a Lymphatic Endothelium Origin for Angiomyolipoma, a TSC2(-) Tumor Related to Lymphangioleiomyomatosis. Am. J. Pathol..

[36] Salic A., Mitchison T.J. (2008). A chemical method for fast and sensitive detection of DNA synthesis in vivo. Proc. Natl. Acad. Sci. USA.

[37] Feoktistova M., Geserick P., Leverkus M. (2016). Crystal Violet Assay for Determining Viability of Cultured Cells. Cold Spring Harb. Protoc..

[38] Oka M., Kobayashi N., Matsumura K., Nishio M., Saeki K. (2019). Exogenous Cytokine-Free Differentiation of Human Pluripotent Stem Cells into Classical Brown Adipocytes. Cells.

[39] Mishina N.M., Bogdanova Y.A., Ermakova Y.G., Panova A.S., Kotova D.A., Bilan D.S., Steinhorn B., Arnér E.S.J., Michel T., Belousov V.V. (2019). Which Antioxidant System Shapes Intracellular H. Antioxid. Redox Signal.

[40] Suthar S.K., Lee S.Y. (2022). Ingenuity pathway analysis of. Front. Mol. Neurosci..

[41] Abdelwahab E.M.M., Bovari-Biri J., Smuk G., Harko T., Fillinger J., Moldvay J., Krymskaya V.P., Pongracz J.E. (2021). Normalization of Enzyme Expression and Activity Regulating Vitamin A Metabolism Increases RAR-Beta Expression and Reduces Cellular Migration and Proliferation in Diseases Caused by Tuberous Sclerosis Gene Mutations. Front. Oncol..

[42] Aydin S. (2015). A short history, principles, and types of ELISA, and our laboratory experience with peptide/protein analyses using ELISA. Peptides.

[43] Zitta K., Meybohm P., Gruenewald M., Cremer J., Zacharowski K.D., Scholz J., Steinfath M., Albrecht M. (2015). Profiling of cell stress protein expression in cardiac tissue of cardiosurgical patients undergoing remote ischemic preconditioning: Implications for thioredoxin in cardioprotection. J. Transl. Med..

[44] Luo C., Ye W.R., Shi W., Yin P., Chen C., He Y.B., Chen M.F., Zu X.B., Cai Y. (2022). Perfect match: mTOR inhibitors and tuberous sclerosis complex. Orphanet J. Rare Dis..

[45] Zhang X., Selvaraju K., Saei A.A., D’Arcy P., Zubarev R.A., Arnér E.S., Linder S. (2019). Repurposing of auranofin: Thioredoxin reductase remains a primary target of the drug. Biochimie.

[46] Zeeshan H.M., Lee G.H., Kim H.R., Chae H.J. (2016). Endoplasmic Reticulum Stress and Associated ROS. Int. J. Mol. Sci..

[47] Nicolussi A., D’Inzeo S., Capalbo C., Giannini G., Coppa A. (2017). The role of peroxiredoxins in cancer. Mol. Clin. Oncol..

[48] Seo M.S., Kang S.W., Kim K., Baines I.C., Lee T.H., Rhee S.G. (2000). Identification of a new type of mammalian peroxiredoxin that forms an intramolecular disulfide as a reaction intermediate. J. Biol. Chem..

[49] Lei H., Wang G., Zhang J., Han Q. (2018). Inhibiting TrxR suppresses liver cancer by inducing apoptosis and eliciting potent antitumor immunity. Oncol. Rep..

[50] Ebrahimi-Fakhari D., Saffari A., Wahlster L., DiNardo A., Turner D., Lewis T.L., Conrad C., Rothberg J.M., Lipton J.O., Kölker S. (2016). Impaired Mitochondrial Dynamics and Mitophagy In Neuronal Models Of Tuberous Sclerosis Complex. Cell Rep..

[51] Feng Z., Levine A.J. (2010). The regulation of energy metabolism and the IGF-1/mTOR pathways by the p53 protein. Trends Cell Biol..

[52] Düvel K., Yecies J.L., Menon S., Raman P., Lipovsky A.I., Souza A.L., Triantafellow E., Ma Q., Gorski R., Cleaver S. (2010). Activation of a metabolic gene regulatory network downstream of mTOR complex 1. Mol. Cell.

[53] Fernández-Vizarra E., Tiranti V., Zeviani M. (2009). Assembly of the oxidative phosphorylation system in humans: What we have learned by studying its defects. Biochim. Biophys. Acta.

[54] Zheng J. (2012). Energy metabolism of cancer: Glycolysis versus oxidative phosphorylation (Review). Oncol. Lett..

[55] Liberti M.V., Locasale J.W. (2016). The Warburg Effect: How Does it Benefit Cancer Cells?. Trends Biochem. Sci..

[56] Hardie D.G., Ross F.A., Hawley S.A. (2012). AMPK: A nutrient and energy sensor that maintains energy homeostasis. Nat. Rev. Mol. Cell Biol..

[57] Kwee J.K. (2014). A paradoxical chemoresistance and tumor suppressive role of antioxidant in solid cancer cells: A strange case of Dr. Jekyll and Mr. Hyde. Biomed. Res. Int..

[58] Knoops B., Goemaere J., Van der Eecken V., Declercq J.P. (2011). Peroxiredoxin 5: Structure, mechanism, and function of the mammalian atypical 2-Cys peroxiredoxin. Antioxid. Redox Signal..

[59] Pylväs M., Puistola U., Kauppila S., Soini Y., Karihtala P. (2010). Oxidative stress-induced antioxidant enzyme expression is an early phenomenon in ovarian carcinogenesis. Eur. J. Cancer.

[60] Bur H., Haapasaari K.M., Turpeenniemi-Hujanen T., Kuittinen O., Auvinen P., Marin K., Koivunen P., Sormunen R., Soini Y., Karihtala P. (2014). Oxidative stress markers and mitochondrial antioxidant enzyme expression are increased in aggressive Hodgkin lymphomas. Histopathology.

[61] Karihtala P., Mäntyniemi A., Kang S.W., Kinnula V.L., Soini Y. (2003). Peroxiredoxins in breast carcinoma. Clin. Cancer Res..

[62] Gérard A.C., Many M.C., Daumerie C., Knoops B., Colin I.M. (2005). Peroxiredoxin 5 expression in the human thyroid gland. Thyroid..

[63] Fernandez-Ranvier G.G., Weng J., Yeh R.F., Shibru D., Khafnashar E., Chung K.W., Hwang J., Duh Q.Y., Clark O.H., Kebebew E. (2008). Candidate diagnostic markers and tumor suppressor genes for adrenocortical carcinoma by expression profile of genes on chromosome 11q13. World J. Surg..

[64] Glasauer A., Chandel N.S. (2014). Targeting antioxidants for cancer therapy. Biochem. Pharmacol..

[65] Gorrini C., Harris I.S., Mak T.W. (2013). Modulation of oxidative stress as an anticancer strategy. Nat. Rev. Drug. Discov..

[66] Bazhin A.V., Philippov P.P., Karakhanova S. (2016). Reactive Oxygen Species in Cancer Biology and Anticancer Therapy. Oxid. Med. Cell Longev..

[67] Wang T., Diaz A.J., Yen Y. (2014). The role of peroxiredoxin II in chemoresistance of breast cancer cells. Breast Cancer.

[68] De Simoni S., Goemaere J., Knoops B. (2008). Silencing of peroxiredoxin 3 and peroxiredoxin 5 reveals the role of mitochondrial peroxiredoxins in the protection of human neuroblastoma SH-SY5Y cells toward MPP+. Neurosci. Lett..

[69] Cao R., Zhang W., Zhang H., Wang L., Chen X., Ren X., Cheng B., Xia J. (2022). Comprehensive Analysis of the PRDXs Family in Head and Neck Squamous Cell Carcinoma. Front. Oncol..

[70] Chen L., Huang C., Yang X., Zhang Q., Chen F. (2018). Prognostic roles of mRNA expression of peroxiredoxins in lung cancer. Onco Targets Ther..

[71] Cao X., Chen X.M., Xiao W.Z., Li B., Zhang B., Wu Q., Xue Q. (2021). ROS--mediated hypomethylation of PRDX5 promotes STAT3 binding and activates the Nrf2 signaling pathway in NSCLC. Int. J. Mol. Med..

[72] He F., Ru X., Wen T. (2020). NRF2, a Transcription Factor for Stress Response and Beyond. Int. J. Mol. Sci..

[73] De Simoni S., Linard D., Hermans E., Knoops B., Goemaere J. (2013). Mitochondrial peroxiredoxin-5 as potential modulator of mitochondria-ER crosstalk in MPP+-induced cell death. J. Neurochem..

[74] Lin J.H., Walter P., Yen T.S. (2008). Endoplasmic reticulum stress in disease pathogenesis. Annu. Rev. Pathol..

[75] Sidrauski C., Walter P. (1997). The transmembrane kinase Ire1p is a site-specific endonuclease that initiates mRNA splicing in the unfolded protein response. Cell.

[76] Nishitoh H., Matsuzawa A., Tobiume K., Saegusa K., Takeda K., Inoue K., Hori S., Kakizuka A., Ichijo H. (2002). ASK1 is essential for endoplasmic reticulum stress-induced neuronal cell death triggered by expanded polyglutamine repeats. Genes. Dev..

[77] Takeda K., Matsuzawa A., Nishitoh H., Ichijo H. (2003). Roles of MAPKKK ASK1 in stress-induced cell death. Cell Struct. Funct..

[78] Tejman-Yarden N., Miyamoto Y., Leitsch D., Santini J., Debnath A., Gut J., McKerrow J.H., Reed S.L., Eckmann L. (2013). A reprofiled drug, auranofin, is effective against metronidazole-resistant Giardia lamblia. Antimicrob. Agents Chemother..

[79] Lewis D., Capell H.A., McNeil C.J., Iqbal M.S., Brown D.H., Smith W.E. (1983). Gold levels produced by treatment with auranofin and sodium aurothiomalate. Ann. Rheum. Dis..

[80] Roder C., Thomson M.J. (2015). Auranofin: Repurposing an old drug for a golden new age. Drugs R&D.

[81] Law B.K. (2005). Rapamycin: An anti-cancer immunosuppressant?. Crit. Rev. Oncol. Hematol..

[82] Food and Drug Administration, Center for Drug Evaluation and Research (CDER) (2013). Guidance for Industry Codevelopment of Two or More New Investigational Drugs for Use in Combination. https://www.fda.gov/files/drugs/published/Codevelopment-of-Two-or-More-New-Investigational-Drugs-for-Use-in-Combination.pdf.

