# Supplementary material for: Prdx5 in the Regulation of Tuberous Sclerosis Complex Mutation-Induced Signaling Mechanisms
Source: Cells. 2023 Jun 24;12(13):1713. doi: 10.3390/cells12131713 (PMC10340296; doi:10.3390/cells12131713)
Supplement: Supplementary file 1 [file cells-12-01713-s001.zip › cells-2433005-supplementary.pdf]

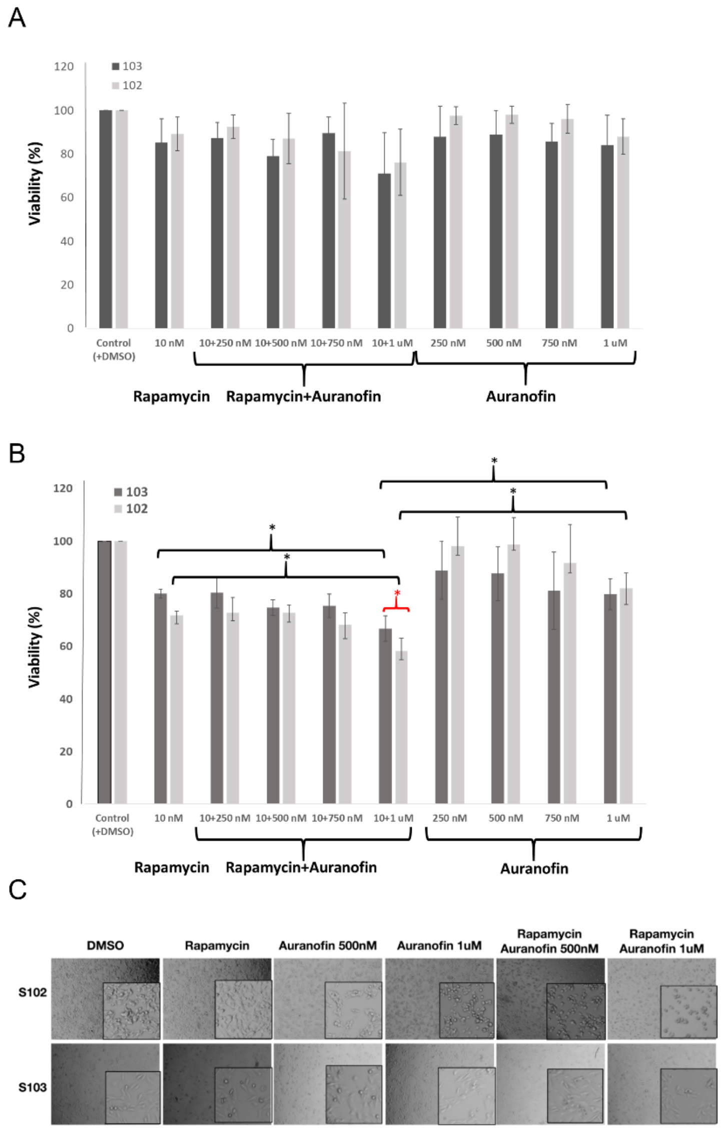

Supplementary Figure S1. Response of S102 and S103 cell lines to rapamycin and auranofin treatment. (**A**) Crystal violet cell viability assay (24 h); (**B)** Crystal violet cell viability assay (48 h). Each treatment was performed in three parallels and repeated five times (n=5). (**C**) Light microscopic images of S102 and S103 cell lines after 10 nM rapamycin, 500 nM and 1 μM auranofin as well as 10 nM rapamycin and 500 nM or 1 μM auranofin combination treatment.

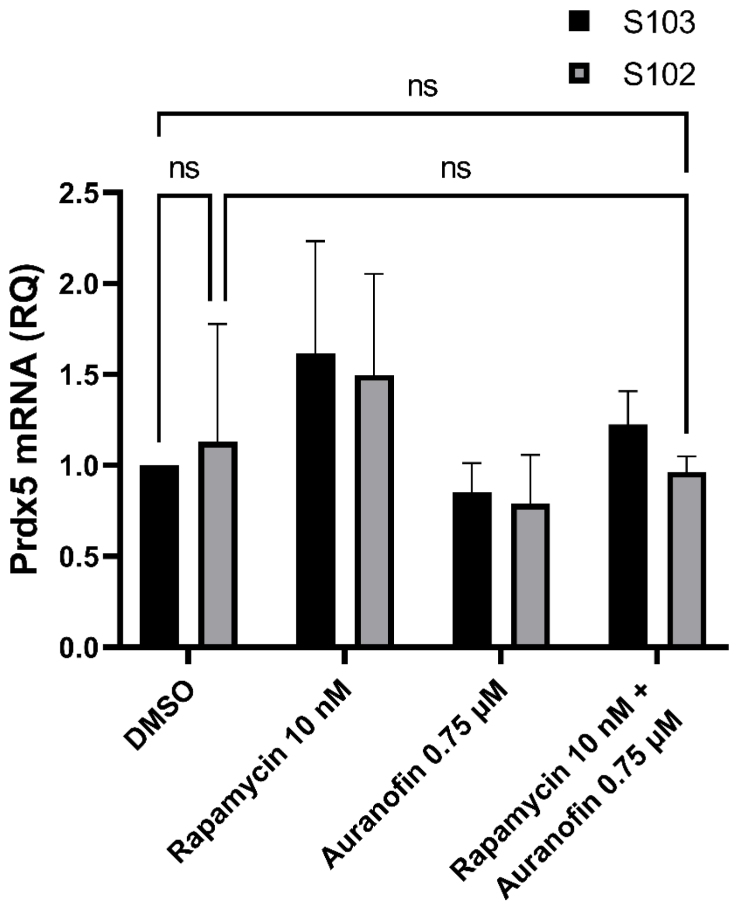

Supplementary Figure S2. Prdx5 gene expression level of TSC WT (S103) and TSC mutant (S102) cells after rapamycin (10 nM) and/or auranofin (0.75 µM) treatment (n=7). Data was calculated and compared to the untreated TSC WT control cell line (S103). Statistically significant values (±SEM) are marked with asterisk (*) ($p<0.0332$).

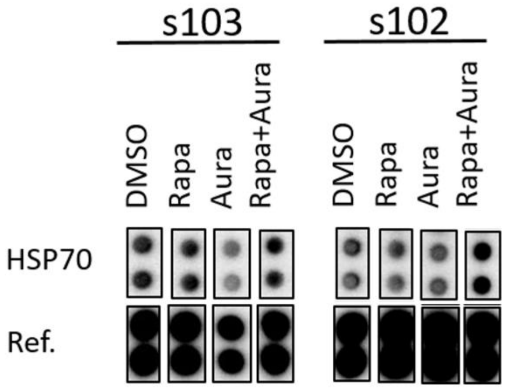

Supplementary Figure S3. Protein expression levels of HSP70 protein of TSC WT (S103) and TSC mutant (S102) cells after rapamycin (10 nM) and/or auranofin (0.75 μM) treatment, measured by Human Proteome Profiler Cell Stress Array.

A

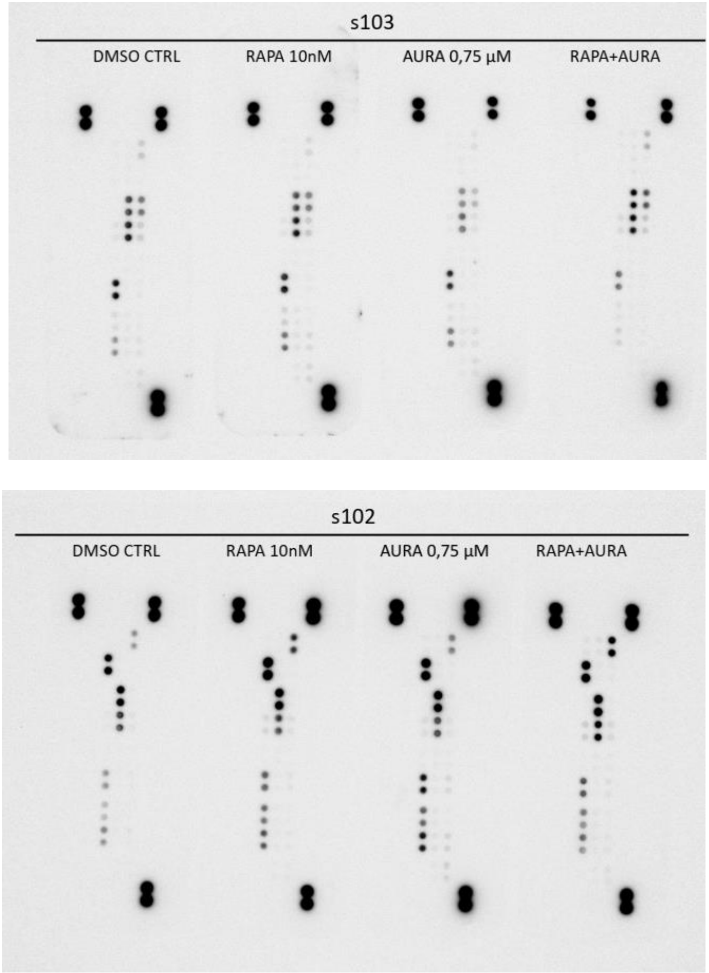

B

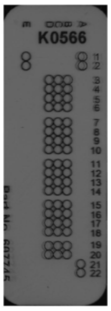

Coordinate	Analyte/Control
A1, A2	Reference Spots
A21, A22	Reference Spots
B3, B4	ADAMTS1
B5, B6	Bcl-2
B7, B8	Carbonic Anhydrase IX
B9, B10	Cited-2
B11, B12	COX-2
B13, B14	Cytochrome c
B15, B16	Dkk-4
B17, B18	FABP-1
B19, B20	HIF-1α
C3, C4	HIF-2α
C5, C6	Phospho-HSP27 (S78/S82)
C7, C8	HSP60
C9, C10	HSP70
C11, C12	IDO
C13, C14	Phospho-JNK Pan (T183/Y185)
C15, C16	NFκB1
C17, C18	p21/CIP1
C19, C20	p27
D3, D4	Phospho-p38α (T180/Y182)
D5, D6	Phospho-p53 (S46)
D7, D8	PON1
D9, D10	PON2
D11, D12	PON3
D13, D14	Thioredoxin-1
D15, D16	SIRT2
D17, D18	SOD2
D19, D20	Negative Control
E1, E2	Reference Spots

Supplementary Figure S4. Cell Stress Array Membranes. (A) Cell stress array membrane of S103 and S102 cells (one representative of n=3), (C) Layout of the Cell Stress Array Membrane.
